# Health-Related Quality of Life Assessment in Patients with Myelodysplastic Syndromes: Evidence from Randomized Clinical Trials

**DOI:** 10.2174/1745017902117010307

**Published:** 2021-12-31

**Authors:** Johannes M. Giesinger, Giorgio La Nasa, Francesco Sparano, Matthias Angermeyer, Emanuela Morelli, Olga Mulas, Fabio Efficace, Giovanni Caocci

**Affiliations:** 1 Medical University of Innsbruck, University Hospital of Psychiatry II, Innsbruck, Austria; 2 Ematologia e CTMO, Ospedale Businco, ARNAS “G. Brotzu”, Cagliari, Italy; 3 Italian Group for Adult Hematologic Diseases (GIMEMA), Data Center and Health Outcomes Research Unit, Rome, Italy; 4 Center for Public Mental Health, University of Vienna, Gosim am Wagram, Austria

**Keywords:** Myelodysplasia, Health related quality of life, IPSS, Erythropoietin, Lenalidomide, HRQoL

## Abstract

Myelodysplastic syndromes (MDS) are characterized by ineffective hematopoiesis and blood cytopenia with a variable risk of progression to acute myeloid leukemia. The main goal of therapy for the large majority of patients is to improve health-related quality of life (HRQoL). Its rigorous assessment is now recommended in international MDS guidelines. Our review provides an overview of HRQoL results from randomized controlled trials (RCTs) in MDS patients. The literature search undertaken in PubMed identified 10 RCTs with HRQoL endpoints (all secondary) published between August 2008 and September 2020. These RCTs have helped to better understand the impact of therapies from the patient perspective and have generated valuable information that can be used to further support clinical decisions. However, the number of RCTs in MDS patients, including HRQoL endpoints, is still low. Given the importance of symptom relief and HRQoL improvement in the treatment of MDS patients, the assessment of the patient perspective in future RCTs is highly recommended to keep expanding the knowledge of the impact of new MDS therapies.

## 1 MYELODYSPLASTIC SYNDROMES AND TREATMENT OPTIONS

Myelodysplastic syndromes (MDS) are characterized by ineffective hematopoiesis and blood cytopenia with a variable risk of progression to acute myeloid leukemia (AML). Chronic anemia, bleeding, and infections represent important complications in MDS that require red blood cell (RBC) and platelet transfusions, as well as supportive treatment with antibiotics and antimycotic drugs [1].

The accurate evaluation of disease risk and progression to AML is crucial for providing patients with an adequate treatment strategy, in particular for those with a poor prognosis. Clinical outcomes in patients with MDS have been evaluated through different scoring systems. The International Prognostic Scoring System (IPSS) is one of the most commonly used prognostic scores in routine practice [2]. This index is based on the percentage of blasts in the bone marrow, cytogenetic abnormalities, and the number of peripheral cytopenias [3]. According to IPSS, the median survival of MDS patients ranges from 6 years for low-risk patients to 6 months for high-risk patients. In 2012, a revised version of IPSS (IPSS-R) based on five rather than three cytogenetic prognostic subgroups and depth of cytopenias, splitting the low marrow blast percentage value, was proposed [4].

Within the last decade, there has been strong evidence that patient-reported outcomes (PROs), including self-reported symptoms, provide prognostic information for survival [5-8]. On this ground, a new MDS prognostic score, which incorporates patient self-reported fatigue severity into the IPSS index, has recently been proposed: the Fatigue FA-IPSS(h) [5]. Specifically, this novel patient-centered risk score has been developed for MDS patients with the higher-risk disease (*i.e*., IPSS intermediate-2 and high-risk) [5] and has also recently been further validated [9]. The FA-IPSS(h) is a pragmatic tool that may further enhance the accuracy of prognosis during the initial diagnostic workup in higher-risk MDS patients.

Currently, several treatment options are available for patients with MDS: hypomethylating agents (*e.g*., azacytidine, decitabine,), hematopoietic stimulating agents (e.g., erythropoietin, G-CSF, luspatercept), supportive care (*e.g*., blood and platelet transfusions, antibiotics), immunomodulatory agents (*e.g*., lenalidomide, cyclosporine, thalidomide), low-dose or intensive chemotherapy, iron overload chelation (deferasirox), and hematopoietic stem cell transplantation (HSCT) [10, 11]. The latter represents the only potentially curative option [12].

However, for many patients, especially the higher-risk MDS patients, the main purpose of therapy is to improve health-related quality of life (HRQoL). Indeed, the drawback of HSCT or chemotherapy is the high risk of treatment-related mortality and morbidity. Hence, it is important that patients are involved by their physicians in the treatment decision-making process to better respond to their needs and preferences [13, 14]. Therefore, the systematic assessment of HRQoL in MDS before and during treatment is recommended.

## 2 HRQoL AND SYMPTOM ASSESSMENT IN PATIENTS WITH MYELODYSPLASTIC SYNDROMES

**Fig. (1):**
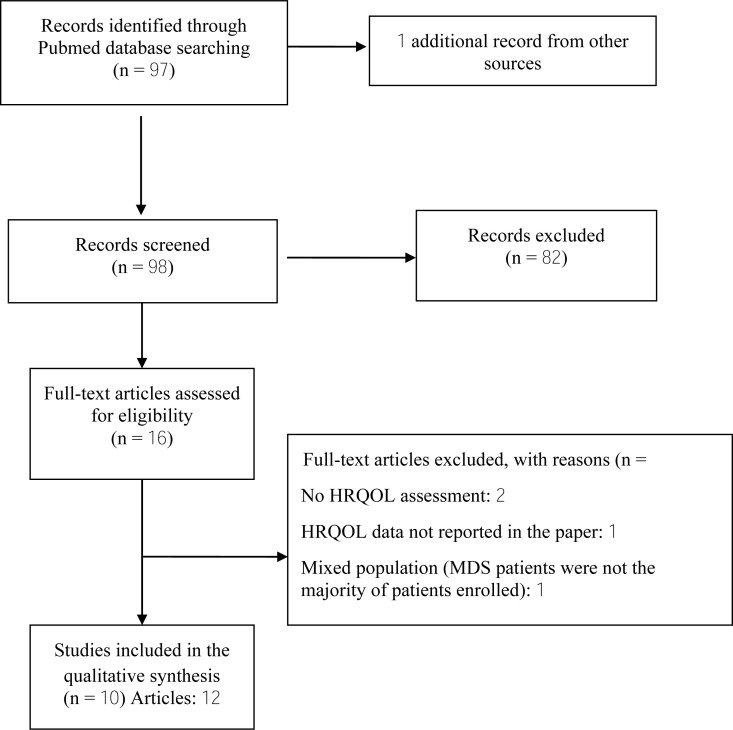
Schematic breakdown of literature search results of MDS randomized controlled trials with HRQOL assessment.

HRQoL is a multidimensional construct for which various definitions are given in the literature (Fig. **1**) [15-18], with most of them covering the patients’ perceptions of the effects of diseases and therapies on the psychological, physical, or social aspects of their life. The importance of this concept builds not only on this broad coverage of possible consequences of disease and treatment but also on its reflection of the patient perspective that gives it a key role in patient-centered care.

Traditionally, in the evaluation of new cancer drugs, clinicians have focused their attention on clinical outcomes such as overall survival, relapse, or response to treatment. However, the U.S. Food and Drug Administration (FDA) has, for over three decades, supported the inclusion of HRQoL assessment along with traditional clinical outcomes in the evaluation of treatment effectiveness [19].

The term “HRQoL” is sometimes confused with the broader term “patient-reported outcome (PRO)” – which refers to “a measurement based on a report that comes directly from the patient […] about the status of a patient’s health condition” [20]. While HRQoL may be considered the most important PRO, this term also covers measurements that are either limited to specific symptoms and treatment side effects or focus on other concepts such as treatment satisfaction and treatment needs.

Unlike other outcomes commonly used in hematology that physicians might measure, such as performance status or treatment toxicity, PROs provide information that originates from the patient directly.

The unique value of PROs is reflected by evidence that indicates that PROs are not inferior to clinician-reported data (*i.e*., performance status) in terms of prognostic value [5, 8].

Standardized PRO measures are available for several onco-hematological diseases. These include generic HRQoL measures (*e.g*., the EORTC QLQ-C30 [21] or Short Form Health Survey used specifically for comparing people with and without onco-hematological diseases [22, 23]; disease-targeted measures (*e.g*., QOL-E [24], QUALMS [25]); dimension-specific measures. Dimension-specific measures (*e.g*., those that focus on the measurement of specific symptoms such as fatigue) may be of particular value in the setting of myelodysplastic syndrome [8]. The considerable number of well-validated PRO measures raises the question of which measure is the most appropriate in MDS patients. The answer depends crucially on the research item of interest and the *a priori* hypothesis about the aspect of HRQoL to be primarily affected by a given treatment [26]. An overview, although not exhaustive, of the principal PRO measures useful in patients with MDS [27] is provided in Table **1**. Notably, for MDS patients, only two disease-specific measures have been developed and validated so far [24, 25]. In recent years, the field of PRO measurement has evolved substantially, resulting in item banks and computer-adaptive assessments that allow for more flexibility in terms of content [28, 29], as well as measurement range and precision [30, 31].

The International Working Group (IWG) has proposed standardized response criteria for the evaluation of clinically relevant responses in MDS, including quality of life (QOL), hematologic improvement, and cytogenetic response [32]. MDS patients are usually elderly and are likely to be affected with one or more comorbidities at the time of diagnosis. Thus, HRQoL can be compromised by several factors. In particular, patients developing anemia require blood transfusions and frequent access to the hospital; moreover, infections and bleeding represent possible complications of neutropenia and thrombocytopenia. One of the most important symptoms in MDS is fatigue, and it is important to note that this symptom is only accessible through patients’ self-reporting [33]. Fatigue is not an isolated symptom but rather a spectrum of symptoms, including physical weakness, lethargy, and reduced mental vigilance [34, 35]. A few studies have properly measured self-reported fatigue, while physicians frequently use the level of anemia as an indicator of fatigue. However, fatigue remains a symptom not entirely correlated to the level of hemoglobin in the blood [33, 36]. Besides the physical symptoms, HRQoL in MDS patients is also influenced by a number of psychosocial and mental factors, such as lack of communication with physicians, lack of understanding of the disease, fear of evolution into acute leukemia, and fear of death [13, 37].

The inclusion of PRO measures in clinical practice might be of value across several hematologic malignancies, as they have a great potential to enable physicians to better monitor patients’ treatment burden and improve HRQoL outcomes [38]. Thus, PRO measurement has acquired considerable weight and occupies a well-earned place alongside the more traditional clinical outcomes. Nevertheless, despite the improvement of the quality of HRQoL assessments in oncology over the past two decades, HRQoL measurement has rarely been included in RCTs in patients with acute leukemias and MDS [39-41]. Therefore, to support the inclusion of HRQoL endpoints in future RCTs, the aim of the following paragraphs is to provide an overview of the RCTs conducted in MDS, which *have* included HRQoL as an endpoint of the study.

## 3 REVIEW OF HRQOL ASSESSMENT IN RANDOMIZED CONTROLLED TRIALS (RCTS) IN PATIENTS WITH MYELODYSPLASTIC SYNDROMES

Since RCTs are the *gold standard* by which healthcare professionals make decisions about treatment efficacy [42], we focused on HRQoL, and symptom outcomes in MDS patients included in RCTs. We previously reported a systematic review of HRQoL studies conducted in MDS patients, which identified 4 RCTs published between January 1980 and July 2008 [39]. Therefore, we mainly aimed at updating this previous work by collecting the most recent evidence stemming from MDS RCTs. For this purpose, a literature search was conducted in PubMed to identify all RCTs published between August 2008 and September 2020. The following search terms were used: (“quality of life” OR “health-related quality of life” OR “health status” OR “health outcomes” OR “patient outcomes” OR “depression” OR “anxiety” OR “emotional” OR “psychosocial” OR “psychological” OR “distress” OR “social” OR “social functioning” OR “social well-being” OR “patient-reported symptom” OR “patient reported outcomes” OR “pain” OR “fatigue” OR “patient-reported outcome” OR “PRO” OR “PROs” OR “HRQL” OR “QOL” OR “HRQoL” OR “symptom distress” OR “symptom burden” OR “symptom assessment” OR “functional status” OR “sexual” OR “functioning”) AND (myelodysplasia OR myelodysplastic syndrome).

All RCTs comparing different conventional treatment modalities and symptom management strategies were considered. No restriction on the number of patients enrolled in the trials was applied. We also considered publications meeting the above criteria and involving heterogeneous patient diseases if MDS patients were the majority of patients enrolled. In total, we screened 98 articles and identified 10 eligible studies (more details are reported in Table **1**. In all the selected RCTs, HRQoL was a secondary endpoint [43-54], *i.e*., no trial used HRQoL as a primary endpoint (Table **2**).

Greenberg *et al*. [43] evaluated the efficacy and long-term safety of Erythropoietin alpha with or without G-CSF plus supportive care (n=53) *versus* standard care alone (n=57) for the treatment of anemic patients with lower-risk MDS. HRQoL was assessed with the Functional Assessment of Cancer Therapy (FACT-G) questionnaire and its fatigue questionnaire module at the time of randomization and at 4 months. In comparison with standard care alone, patients receiving Erythropoietin with or without G-CSF plus supportive care had improved erythroid responses but similar survival and incidence of AML transformation. There were no significant differences in FACT-G scores and fatigue scores between those assigned to Erythropoietin and those assigned to standard care; however, patients with an erythroid response at 4 months reported a significant improvement from baseline in physical, emotional, and functional well-being, as well as fatigue and overall HRQoL.

Lubbert *et al*. [44] compared low-dose Decitabine to best supportive care in higher-risk MDS patients older than 60 years who were ineligible for intensive chemotherapy. Decitabine was given three times a day for 3 days in 6-week cycles. Overall survival was not different between the two arms, while progression-free survival was significantly prolonged in the Decitabine arm. Assessment of HRQoL with the EORTC QLQ-C30 showed a significant improvement in fatigue and physical functioning in patients treated with Decitabine.

In the MDS-004 trial [45, 46], the efficacy and safety of Lenalidomide (10 or 5 mg on days 1-21) *versus* placebo was assessed in 205 red blood cell transfusion-dependent patients with IPSS Low-Intermediate-1-risk del5q31 MDS. More patients in the lenalidomide 10 and 5 mg groups achieved red blood cells transfusion independence for > 26 weeks (primary endpoint) compared to the placebo arm. HRQoL was assessed using the Functional Assessment of Cancer Therapy-Anemia (FACT-An) questionnaire, administered at baseline and at 12, 24, 36, and 48 weeks. Mean change from baseline at week 12 in the FACT-An total score was significantly higher for patients treated with Lenalidomide 10 mg and 5 mg compared to patients receiving placebo. Also, a clinically meaningful improvement in FACT-An scores was seen in patients who switched from the placebo group to the lenalidomide 5 mg group after week 12.

In phase III MDS-005 study by Santini *et al*. [47, 48], the authors evaluated HRQoL among red blood cell transfusion-dependent patients with lower-risk non-del(5q) MDS treated with Lenalidomide (n=160) or placebo (n=79). HRQoL, a predefined secondary endpoint, was assessed using the EORTC QLQ-C30 questionnaire at baseline, week 12, week 24, every 12 weeks thereafter, and at discontinuation. At week 24, lenalidomide was associated with benefits over placebo across all five preselected questionnaire scales (fatigue, dyspnea, global QOL, physical functioning, and emotional functioning).

Yu *et al*. [49] compared the outcomes of 91 low-intermediate risk MDS patients treated with supportive care and chemotherapy to those of 91 patients treated with allogeneic HSCT. The complete remission rate and overall survival in the allogeneic HSCT group were significantly better than in the control group. The EORTC QLQ-C30 was used to assess HRQoL at four-time points (baseline, and 3, 6, and 12 months after HSCT). The HSCT patients’ physical and social functioning was significantly more improved at follow-up than in the control group.

Oliva *et al*. [50] evaluated the efficacy and safety of Eltrombopag, a thrombopoietin agonist, versus placebo in a single-blind, phase 2 superiority RCT in adult patients with low-risk or IPSS intermediate-1-risk MDS and severe thrombocytopenia. Platelet responses occurred in 47% of patients in the Eltrombopag group versus 3% of patients in the placebo group. The outcome events AML evolution or disease progression occurred in 12% of patients in the Eltrombopag group versus 16% of patients in the placebo group. HRQoL was assessed longitudinally (at baseline and every 3 months) with the EORTC QLQ-C30 and the QOL-E. The authors did not find significant changes over time in the QOL-E items and accordingly no significant difference between groups but found that QOL-E scores were better in patients with higher platelet counts.

Platzbecker *et al*. [53] assessed the efficacy and safety of subcutaneous darbepoetin alfa in 147 patients with IPSS low/intermediate-1 risk MDS, anemia, and low transfusion burden. The authors found that 24 weeks of darbepoetin alfa significantly reduced transfusion incidence (36.1% versus 59.2% in the placebo group) and increased erythroid response rates (14.7% versus 0% in the placebo group). PRO data showed no differences between treatment arms. Changes from baseline to week 24 measured with the EQ-5D VAS did not differ between groups, nor were clinically meaningful differences found for FACIT-Fatigue scores. As highlighted by the authors, the study was not powered for PRO analyses, and the lack of statistically significant improvements in PROs may be a result of the small number of treatment responders.

Similar results were described in a study conducted by Fenaux *et al*. [51], which evaluated the efficacy and safety of epoetin-α in 130 anemic patients with low-risk MDS. Compared with placebo, an epoetin-α improved erythroid response, reduced the percentage of patients requiring RBC transfusion, and increased time to the first transfusion. As in the study conducted by Platzbecker *et al*. [53], the clinical improvements were not accompanied by significant differences in PROs, measured with the FACT-An and the EQ-5D-3L. No differences between epoetin-α and placebo were found at any time point, except at week 24 when the EQ-5D index score was significantly different between groups.

Sanchez-Garcia *et al*. [52] compared azacitidine and best supportive care in 40 patients with low-risk MDS without del(5q). A significantly higher erythroid response rate was found in patients randomized to azacitidine (44.5%) compared to those receiving the best supportive care (5.5%). PRO results, assessed with the FACT-An questionnaire, showed no statistically significant differences between study arms and no improvements 9 months after randomization.

Finally, Stanworth *et al*. [54] compared two different red cell thresholds for transfusion in 38 MDS patients: a restrictive transfusion strategy to maintain a hemoglobin concentration between 85 and 100 g/l *versus* a liberal transfusion strategy to maintain the hemoglobin concentration between 110 and 125 g/l. HRQoL was assessed using the EORTC QLQ-C30 and the EQ-5D-5L. The number of patients achieving a clinically meaningful increase was higher in the liberal arm across the EQ-5D-5L score and the fatigue and global health score domains of the EORTC QLQ-C30. An exploratory analysis showed that higher hemoglobin thresholds might be associated with improved HRQoL.

## 4 DISCUSSION

We illustrated the progress of HRQoL research in patients with MDS by investigating the results of RCTs with HRQoL endpoints. While in our previous systematic review, we only found 4 RCTs with HRQoL published between 1980 and 2008 [39], the current review identified that 10 had been published since 2008. This result may reflect a recently increasing interest in HRQoL research in MDS patients. It should also be noted that the results of a phase III RCT comparing luspatercept versus placebo in IPSS-R-defined lower-risk MDS patients have been recently published [55]. HRQoL, measured with the EORTC QLQ-C30, was a secondary endpoint of this study, but since the results for this endpoint have not yet been published, this study was not included in our review. The relatively low number of studies in this area denotes the need to further improve the use of PROs in MDS patients. As even aggressive chemotherapy and HSCT offer only low chances of survival, the physician’s general perception may be that it could be difficult to assess the patient perspective in a clinical trial setting [56]. For example, according to a cross-sectional survey of 180 physicians, 72 percent of them said their patients were willing to accept poor HRQoL in exchange for a small chance of cure, 47 percent said they did not use HRQoL data, and 55 percent said they would be more likely to use HRQoL data if it was more understandable [57]. While asking patients directly may result in a different balance of HRQoL vs. chance of cure, these findings may in part explain the limited number of HRQoL studies in MDS patients.

## CONCLUSION

Overall, the HRQoL results stemming from the selected trials provide unique information on the symptom burden perceived by the patient and the effects of treatment on their HRQoL. Systematic inclusion of HRQoL assessment in clinical studies is critical for providing both clinicians and patients with a more comprehensive understanding of the overall treatment effectiveness. Fatigue assessment represents an important endpoint that is significantly associated with impairments of HRQoL and the ability to work or participate in desired activities. Commonly assessed hemoglobin levels cannot fully explain patient-reported fatigue, and several studies have found a weak correlation between anemia and chronic fatigue in MDS [58-68]. Since alleviation of disease-related complications and improved HRQoL are essential goals of MDS treatment, the systematic assessment of HRQoL parameters in clinical trials should be considered for inclusion as endpoints in future studies. Over the last years, major improvements in HRQoL research in MDS patients have been made. For example, two PRO measures have been recently developed specifically for MDS patients, namely the QOL-E [24] and the QUALMS [25], which may capture some disease-specific symptoms and impairments better than generic cancer instruments such as the FACT-G or EORTC QLQ-C30. It is hoped that the growing interest in investigating HRQoL in MDS patients will further encourage the inclusion of PROs in this setting in order to improve the quality of PRO data that can help patients and clinicians when making treatment decisions. Therefore, the implementation of HRQoL assessment in future studies is highly recommended to continue expanding knowledge of the impact of MDS therapies.

## Figures and Tables

**Table 1:** Selected Patient-Reported Outcome questionnaires that can be used in patients with MDS.

**GENERIC AND CANCER-SPECIFIC HRQoL INSTRUMENTS**	**MDS- AND SYMPTOM-SPECIFIC INSTRUMENTS**
***GENERIC HRQoL INSTRUMENTS***	***MYELODYSPLASTIC SYNDROMES***
EuroQol 5 Dimensions (EQ-5D) [59]	Quality of Life in Myelodysplasia Scale (QUALMS) [25]
Short Form 36 Health Survey (SF-36) [60]	Quality of Life E (QOL-E) [24]
***CANCER-SPECIFIC HRQoL INSTRUMENTS***	***FATIGUE***
EORTC Quality of Life Questionnaire-Core 30 (EORTC QLQ-C30) [21]	Functional Assessment of Chronic Illness Therapy – Fatigue (FACIT-Fatigue) [61]
Functional Assessment of Cancer Therapy-General (FACT-G) [62]	FACT-Anemia (FACT-An) [63]
	Brief Fatigue Inventory [64]
	Fatigue Symptom Inventory [65]
	Multidimensional Fatigue Inventory [66]
	***MENTAL DIMENSION***
	Hospital Anxiety and Depression Scale (HADS) [67]
	Memorial Symptom Assessment Scale (MSAS) [68]

Abbreviations: EORTC = European Organisation for Research and Treatment of Cancer; HRQoL = health-related quality of life; MDS = myelodysplastic syndromes.

**Table 2:** Summary of Clinical and HRQoL outcomes of RCTs in patients with MDS.

**Authors**	**Year**	**N° of Patients Randomized**	**HRQoL Endpoint**	**PRO Instruments**	**Summary of Clinical Outcomes**	**Summary of HRQoL Outcomes**
Greenberg *et al*. [43]	2009	110	Secondary	FACT-G, FACIT Fatigue	Erythroid independence rate was higher in patients treated with Erythropoietin in comparison with best supportive care	Patients with erythroid independence reported significant improvement from the baseline in physical, emotional, and functional well-being, as well as in fatigue and overall quality of life
Lubbert *et al*. [44]	2011	233	Secondary	EORTC QLQ-C30	Progression-free survival was higher in patients treated with Decitabine in comparison with best supportive care	Improvement in fatigue and physical functioning in patients treated with Decitabine
Fenaux *et al*.; Revicki *et al*. [45, 46]	2011	205	Secondary	FACT-An	The erythroid response was higher in the Lenalidomide arm in comparison with the placebo	Fatigue was significantly improved in Lenalidomide arms than in placebo
Santini *et al*. [47, 48]	2016	239	Secondary	EORTC QLQ-C30	Sustained red blood cell response in patients treated with lenalidomide in comparison with placebo	Response to lenalidomide was associated with improved fatigue, dyspnea, global quality of life, physical functioning, and emotional functioning
Yu *et al*. [49]	2017	182	Secondary	EORTC QLQ-C30	Survival was higher in transplanted patients in comparison with conventional chemotherapy	Improvement of HRQoL in patients who underwent hematopoietic stem cell transplantation
Oliva *et al*. [50]	2017	90	Secondary	EORTC QLQ-C30 QOL-E	Higher platelet responses occurred in patients treated with Eltrombopag in comparison with placebo	QOL-E social, sexual, MDS-specific, treatment outcome index, general, and all scores improved with increasing platelet counts
Platzbecker *et al*. [53]	2017	147	Secondary	FACIT-Fatigue EQ-5D VAS	Transfusion incidence was lower with darbepoetin alfa versus placebo and erythroid response rates increased with darbepoetin alfa	No differences between darbepoetin alfa and placebo were found.
Fenaux *et al*. [51]	2018	130	Secondary	FACT-An EQ-5D-3L	Epoetin-α improved erythroid response reduced the percentage of patients requiring red blood cell transfusion and increased the time to the first transfusion compared with placebo	There were no differences in QOL between the epoetin-α group and the placebo at any time point. QOL at week 24 was different between the responders in the epoetin-α group and the placebo group
Sanchez-Garcia *et al*. [52]	2018	40	Secondary	FACT-An	Erythroid hematologic improvement was higher in patients randomized to azacytidine than in those receiving the best supportive care	No differences between arms were found. FACT-An scores did not show improvements from baseline
Stanworth *et al*. [54]	2020	38	Secondary	EQ-5D-5L EORTC QLQ-C30	The proportion of compliance to treatment threshold was ≥ 70% in both arms, and the study was declared feasible	The number of participants achieving a (pre-defined) clinically meaningful increase showed small improvements favoring the liberal policy across the following domains (EQ-5D-5L descriptive; EORTC QLQ-C30: fatigue and global health score)

Abbreviations: EORTC = European Organisation for Research and Treatment of Cancer; FACT = Functional Assessment of Cancer Therapy; HRQoL = health-related quality of life; MDS = myelodysplastic syndromes; PRO = patient-reported outcomes; QLQ-C30 = Quality of Life questionnaire-Core 30; QOL = quality of life; RCT = randomized controlled trial; VAS = visual analogue scale.
